# Co-localization of the sodium-glucose co-transporter-2 channel (SGLT-2) with endothelin ET_A_ and ET_B_ receptors in human cardiorenal tissue

**DOI:** 10.1042/BSR20240604

**Published:** 2024-05-31

**Authors:** Thomas L. Williams, Rhoda E. Kuc, Anna L. Paterson, George R. Abraham, Anna L. Pullinger, Janet J. Maguire, Sanjay Sinha, Peter J. Greasley, Philip Ambery, Anthony P. Davenport

**Affiliations:** 1 Division of Experimental Medicine and Immunotherapeutics, University of Cambridge, Addenbrooke's Hospital, Cambridge U.K.; 2 Department of Pathology, Cambridge University Hospitals NHS Foundation Trust, Cambridge, U.K.; 3 Royal Papworth Hospital NHS Foundation Trust, Cambridge Biomedical Campus, Cambridge, U.K.; 4 Wellcome-MRC Cambridge Stem Cell Institute, Jeffrey Cheah Biomedical Centre, University of Cambridge, Cambridge, U.K.; 5 Early Clinical Development, Research and Early Development, Cardiovascular, Renal and Metabolism (CVRM), BioPharmaceuticals R&D, AstraZeneca, Gothenburg, Sweden; 6 Late-Stage Development, Cardiovascular, Renal and Metabolism, BioPharmaceuticals R&D, AstraZeneca, Gothenburg, Sweden

**Keywords:** Endothelin-1, SGLT-2, sodium-glucose co-transporter-2, sodium-glucose co-transporter-2 inhibitors

## Abstract

Endothelin (ET) receptor antagonists are being investigated in combination with sodium-glucose co-transporter-2 inhibitors (SGLT-2i). These drugs primarily inhibit the SGLT-2 transporter that, in humans, is thought to be mainly restricted to the renal proximal convoluted tubule, resulting in increased glucose excretion favouring improved glycaemic control and diuresis. This action reduces fluid retention with ET receptor antagonists. Studies have suggested SGLT-2 may also be expressed in cardiomyocytes of human heart. To understand the potential of combining the two classes of drugs, our aim was to compare the distribution of ET receptor sub-types in human kidney, with SGLT-2. Secondly, using the same experimental conditions, we determined if SGLT-2 expression could be detected in human heart and whether the transporter co-localised with ET receptors.

Methods: Immunocytochemistry localised SGLT-2, ET_A_ and ET_B_ receptors in sections of histologically normal kidney, left ventricle from patients undergoing heart transplantation or controls. Primary antisera were visualised using fluorescent microscopy. Image analysis was used to measure intensity compared with background in adjacent control sections.

Results: As expected, SGLT-2 localised to epithelial cells of the proximal convoluted tubules, and co-localised with both ET receptor sub-types. Similarly, ET_A_ receptors predominated in cardiomyocytes; low (compared with kidney but above background) positive staining was also detected for SGLT-2.

Discussion: Whether low levels of SGLT-2 have a (patho)physiological role in cardiomyocytes is not known but results suggest the effect of direct blockade of sodium (and glucose) influx via SGLT-2 inhibition in cardiomyocytes should be explored, with potential for additive effects with ET_A_ antagonists.

## Introduction

Endothelin (ET) receptor antagonists are being investigated in combination with sodium-glucose co-transporter-2 inhibitors (SGLT-2i) for a variety of clinical conditions [1–3]. The benefit of combining these two drugs was initially suggested following post-hoc analysis of the pivotal SONAR trial in chronic kidney disease [4]. The SGLT-2 transporter in humans, is thought to be mainly restricted to the renal proximal convoluted tubule, resulting in increased glucose excretion favouring improved glycaemic control in Type 2 Diabetes (T2DM) and diuresis. SGLT-2i co-therapy may reduce fluid retention observed with ET receptor antagonists, a mechanism of action demonstrated in an animal model using haematocrit and bodyweight [5]. In recent years, a wealth of evidence has accumulated for SGLT-2i and dramatic decreases in cardiovascular adverse event rates, independent of glycaemic status, resulting in landmark trials in heart failure [6]. Multiple mechanisms of action for SGLT-2i remain under investigation to explain the efficacy demonstrated in heart failure [7].

Studies have suggested SGLT-2 may also be expressed in cardiomyocytes of human heart [8]. For example, the gene encoding SGLT-2 has been identified in human ventricular cardiomyocytes using single cell RNA-Seq [9] and in cardiomyocytes derived from human induced pluripotent stem cells [10]. In functional experiments *in vitro*, using AC16 cells that express markers characteristic of cardiomyocytes, Scisciola et al. [11] proposed that an SGLT-2i mainly acting on SGLT-2, had beneficial actions in preventing epigenetic (DNA methylation) changes induced by high glucose.

To understand the potential of combining SGLT-2i and endothelin antagonists, our aim was to compare the distribution of ET receptor sub-types in human kidney, with SGLT-2. Secondly, using the same experimental conditions we determined if SGLT-2 expression could be detected in human heart and whether the transporter co-localised with ET receptors, which has not previously been determined. In order to identify a clinically relevant model for *in vitro* characterisation, we demonstrate the expression of SGLT-2 in cultures of beating human embryonic stem cell derived cardiomyocytes. The precise determination of the cellular localization of receptors and transporters is critical for understanding the mechanism of action of drugs designed to block their action, particularly when used in combination, as is the case of ET antagonists and SGLT-2 inhibitors.

## Methods

### Immunocytochemistry

Immunocytochemistry was performed to localise SGLT-2, ET_A_ and ET_B_ receptors in fresh frozen cryostat sections (10 µm, prepared and stored at −70°C) of surgical samples of human tissue (*n* = 3 individuals in triplicate) obtained with written informed consent and ethical approval (REC 05/Q104/142). Histologically normal kidney removed after nephrectomy for carcinoma, left ventricle from patients undergoing heart transplantation for dilated cardiomyopathy (DCM) or control (C) tissue from hearts considered suitable for heart transplantation but where no suitable recipient could be matched.

Tissue sections were thawed and rehydrated in phosphate buffered saine (PBS), fixed for 3 to 5 min in paraformaldehyde solution (4% buffered, pH 6.9, 1.00496.8350; Sigma-Aldrich) and then washed 3×5 min with PBS. A very short exposure to paraformaldehyde solution was used to minimize autofluorescence in tissues. Non-specific staining was blocked by incubating with PBS containing 10% donkey sera, for 2 h at room temperature. Tissue sections were incubated overnight at 4°C with a panel of primary antibodies raised in mouse against SGLT-2 (Abcam; ab58298; amino acid sequences 228-278, diluted at 1:50 / 1:100 / 1:300 and Santa Cruz; SC-393350; amino acid sequences 220-270, diluted at 1:50) as part of the validation process as it is unlikely that two antisera to different epitopes would give the same pattern of distribution by chance. Both produced a single band in Western blots of the expected size and gave the same distribution in tissue. The Abcam (ab58298) antibody was selected for further experiments throughout the study because it was raised in mouse and could be used to co-localise with ET_A_ and ET_B_ receptor antibodies raised in rabbit without any cross-reactivity.

Primary antibodies for ET_A_ were raised in rabbit (AER_001; Alomone; diluted at 1:100, against the sequence 413-426, giving a single band in Western blot). ET_B_ antisera were also raised in rabbit (Rb D51B; in-house; sequence 428-442, diluted at 1:50,) and previously validated by absence of staining in endothelial cell-specific ET_B_ knockout mice [12]. Antibodies for cellular markers were to human smooth muscle α-actin (M0851; Dako; 1:100) and human Von Willebrand Factor (vWF; M0616; Dako; 1:50). All primary and secondary antibodies were prepared in a diluent consisting of PBS with 1% donkey serum, 0.1% Tween-20, and 3.3 mg/ml bovine serum albumin. Buffer only controls, omitting the primary antibodies, were incubated with diluent alone. All slides were subsequently washed 3× with PBS with 0.1% Tween-20 (PBS/T) before incubation for 1 h at room temperature in the dark with fluorescently conjugated secondary antisera: polyclonal donkey anti-rabbit IgG H&L antibody conjugated to Alexa Fluor 488 (ab150061; Abcam; 1:200); donkey anti-mouse IgG H&L antibody conjugated to Alexa Fluor 555 (ab150110; Abcam; 1:200) prepared in diluent as above. Slides were again washed 3× with PBS/T before incubation with Hoechst 33342 nuclear stain (H3570; Invitrogen) prepared at 10 μg/ml in diluent as above, for 20 min at room temperature in the dark. Following a final 3× washes with PBS/T, slides were blotted dry with lint-free tissue, mounted with ProLong Gold Antifade Mountant, covered with a cover slip, and left at room temperature in the dark to cure (≥48 h).

### Culture of human embryonic stem cells derived cardiomyocytes

RUES2 human embryonic stem cells were maintained in culture and induced to differentiate to beating cardiomyocytes (hESC-CMs) using a previously optimised protocol [13] based on manipulation of the Wnt signalling pathway. Differentiation to mesoderm was induced in hESCs (at a density of 2 × 10^6^ cells/well) on Matrigel (orning, 354277) coated 6-well plates by culturing in RPMI media (Gibco, 21875-034) supplemented with the small molecule Wnt activator CHIR99021 (6uM, Selleckchem, S2924). After 24 h, an additional 2 ml RPMI media was added to dilute supplemented CHIR99021. Media was removed and replaced with RPMI only after an additional 24 h at 37°C. Twenty-four hours later, media was replaced with RPMI supplemented with the Wnt pathway inhibitor Wnt C59 (2.5 μM, Stratech, S7037) to direct mesoderm progenitors towards cardiac mesoderm cells. After 48 h, media was replaced with RPMI supplemented with B27 minus insulin (Fisher Scientific, A1895601). Forty-eight hours later, media was changed to RPMI supplemented with B27 plus insulin (Fisher Scientific, 17504044) and maintained until robust beating was established (around day 12 of differentiation). Immunocytochemistry to determine expression of SGLT-2, ET_A_ and ET_B_ was carried out as above.

### Fluorescent imaging and whole slide digitisation

Automated fluorescent images (16 bit, 0.325 × 0.325 μm scaling per pixel) of fixed and mounted human tissue sections stained with fluorescent markers were acquired using an Axio Scan Z1 (Zeiss) slide scanner with a Plan-Apochromat 20x/NA0.8 M27 objective lens connected to a Hamamatsu Orca Flash camera. This is an automated microscope system. Multiple slides are loaded and digitised at the same time to capture the entire specimen area of the slide. The sensitive camera and maximally corrected optics achieve optimal image quality and the UV-free LED light source and a focus finder with oblique illumination, ensure maximum protection for the sample. An initial brightfield scan was performed to visualise tissue on slides, and a spline contour tool was used to outline the tissue to minimise the total region imaged. A profile with three fluorescent channels was used; a first channel (blue), with an LED-Module 385 nm light source set at 10% intensity and 10 ms exposure time at a depth of focus of 1.45 μm for Hoechst 33342 nuclear marker (405 nm wavelength); a second channel (green), with an LED-Module 475 nm light source set at 80% intensity and 30 ms exposure time at a depth of focus of 1.64 μm for 488 nm wavelengths; and a third channel (yellow/orange), an LED-Module 567 nm light source set at 80% intensity and 30 ms exposure time at a depth of focus of 1.88 μm for 555 nm wavelengths. The short exposure times were used to both reduce bleaching of the added fluorophores and reduce observable autofluorescence to improve signal-to-noise. The scanner is very sensitive in detecting fluorescence and LED light sources ensure low and consistent illumination to minimise autofluorescent signal. The whole image of the slide is captured automatically, without operator intervention. All slides used in the study were loaded into slide scanner and imaged at the same time, using identical settings, using the same predetermined profile. Focal depths were determined by the slide scanner’s in-built Z stacking autofocusing. All acquired images comprising the entire microscope slide, were saved and visualised using ZEN software (Zeiss) and/or Orbit Image Analysis (ORBIT) software. Regions of interest were identified using the spline contour, and mean fluorescent signal (in grayscales) was provided by ZEN software (Zeiss) [14,15]. Equivalent sections were imaged in adjacent control sections where primary antisera were omitted but processed at the same time in otherwise identical conditions to measure background levels of fluorescence. Significant differences in fluorescence intensity (grayscales) between the test samples and adjacent controls indicated positive staining and expression of the target protein. The post-acquisition analyses of the digital image were also identical between all positive samples and the negative controls. Differences were significant if *P*<0.05. We did not compare relative levels of staining for different protein targets eg ET_A_ versus ET_B_ as the polyclonal antibodies used may have different affinities, leading to variable amplification of signal.

Fluorescent confocal images of hESC-CMs were acquired using the Opera Phenix High Content Screening System (PerkinElmer) microscope with a 40×/NA1.1 water immersion objective. Excitation/emission laser and filter sets for three fluorescent channels were used: 405/435–480 nm (blue), 488/500–550 nm (green), and 561/570–630 nm (gold). Laser intensities for all channels were set to 50% transmission, with a 50 ms exposure time. Opera Phenix hardware focus ensures a consistent focal depth across the CellCarrier-96 Ultra Plate is achieved. Scale bars show 50 μm unless specified otherwise.

## Results

### Human kidney control

**Figure 1:**
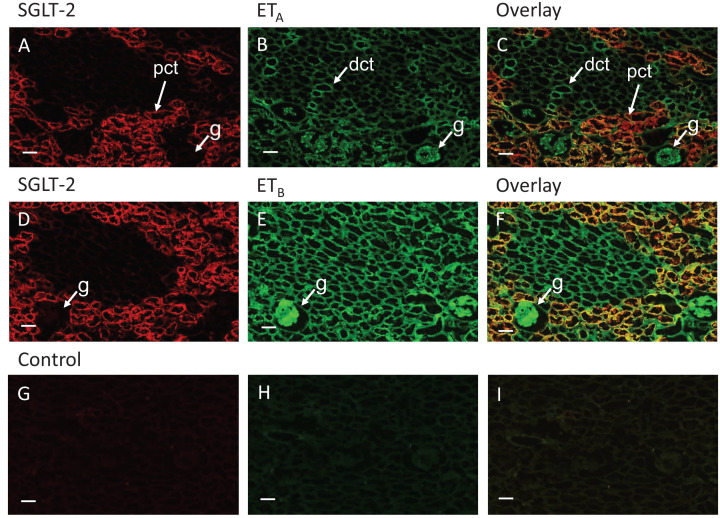
SGLT-2, ET_A_ and ET_B_ distribution in reference human kidney tissue Representative fluorescent images of sections of human kidney following dual labelling with antisera to SGLT-2, visualised in red (**A,D**) and visualised in green ET_A_ (**B**) or ET_B_ (**E**). Overlay showing co-localization in epithelial cells of the proximal convoluted tubules (visualised in yellow). (**G**–**I**) Adjacent sections where the primary antisera have been omitted to show levels of background fluorescence. (pct, proximal convoluted tubules, dct, distal convoluted tubules, g, glomeruli, scale bar = 110 μm).

**Figure 2:**
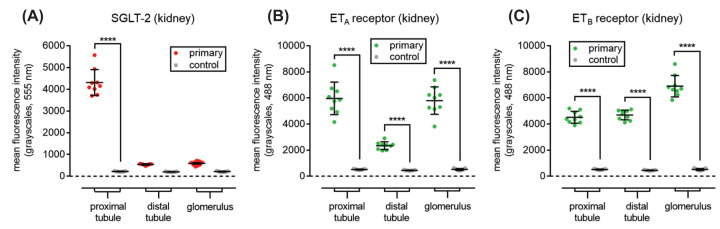
Quantification of SGLT-2, ET_A_ and ET_B_ immunofluorescence in kidney regions Bar charts show quantitative 555 nm fluorescence (grayscales) for kidney structures stained with SGLT-2 antisera and quantitative 488 nm fluorescence (grayscales) for kidney structures stained with ET_A_ and ET_B_ antisera compared with control sections. Data are expressed as mean ± s.e.m. *n* = 3 individuals, in triplicate. Statistical significance was determined using a one-way ANOVA, with Tukey’s correction for multiple comparisons for differences between treatment groups; ****P<0.0001.

In the reference tissue, the human kidney, SGLT-2 immunoreactivity displayed the expected restricted distribution and localised to the epithelial cells of the proximal convoluted tubules (Figure 1A,D). The intensity of fluorescence was significantly higher than control sections (Figure 1G), where the primary antisera was omitted (Figure 2A). Antisera to ET_A_ receptor (Figure 1B) also showed staining of the epithelial cells of the proximal convoluted tubules and co-localised with SGLT-2 in the overlay (yellow, Figure 1C). As expected, ET_A_ staining also localised to a sub-population of epithelial cells in the distal convoluted tubules and mesangial cells of the glomerulus (Figure 1B). ET_B_ receptor immunoreactivity (Figure 1F) also co-localised with SGLT-2 in proximal epithelial cells. Fluorescence for ET_B_ was also visualised as expected (Figure 1E) in distal tubule epithelial cells, endothelium of intra-renal vessels and in the glomeruli. Quantification of SGLT-2, ET_A_ and ET_B_ immunofluorescence in kidney regions is shown in (Figure 2).

### Human heart dilated cardiomyopathy

**Figure 3:**
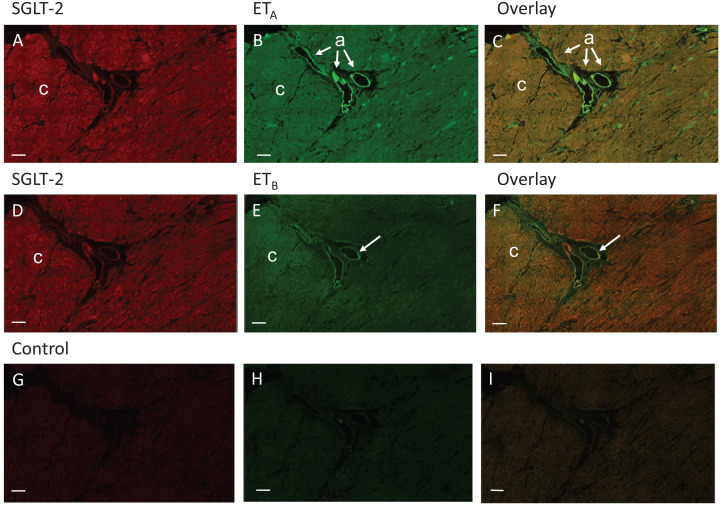
SGLT-2, ET_A_ and ET_B_ distribution in DCM human heart - left ventricle tissue Representative fluorescent images of sections of left ventricle from DCM heart following dual labelling with antisera to SGLT-2 (**A,D** red) and ET_A/B_ receptor subtypes (**B,E**, green) to cardiomyocytes and smooth muscle of blood vessels (indicated in B and C by arrows labelled a for arterioles). ET_A_ receptors on smooth muscle were visualised in green (B) with lower levels to cardiomyocytes that colocalised with SGLT-2 in the overlay (**C**). SGLT-2 fluorescence (D) also colocalized to low densities of ET_B_ receptors (E) present on cardiomyocytes, shown in the overlay (**F**). ET_B_ staining was detected in endothelial cells in (E and F), indicated by arrows. (**G**–**I)** Adjacent sections where the primary antisera have been omitted to show levels of background fluorescence. In (A–F), examples of cardiomyocytes within ventricular free wall are indicated by c., scale bar = 300 μm).

In the same assay as the reference kidney tissue, low levels (compared with kidney but above background measure in the negative control) of positive staining were detected for SGLT-2 in cardiomyocytes of the left ventricle in both control and DCM hearts. In both cases, as expected, ET_A_ staining was most intense on the vascular smooth muscle with lower levels present in the cardiomyocytes, colocalising with SGLT-2 (Figure 3A–C). A similar pattern of co-expression with SGLT-2 to cardiomyocytes was seen for ET_B_ (Figure 3D–F) although staining was less than for ET_A_.

**Figure 4:**
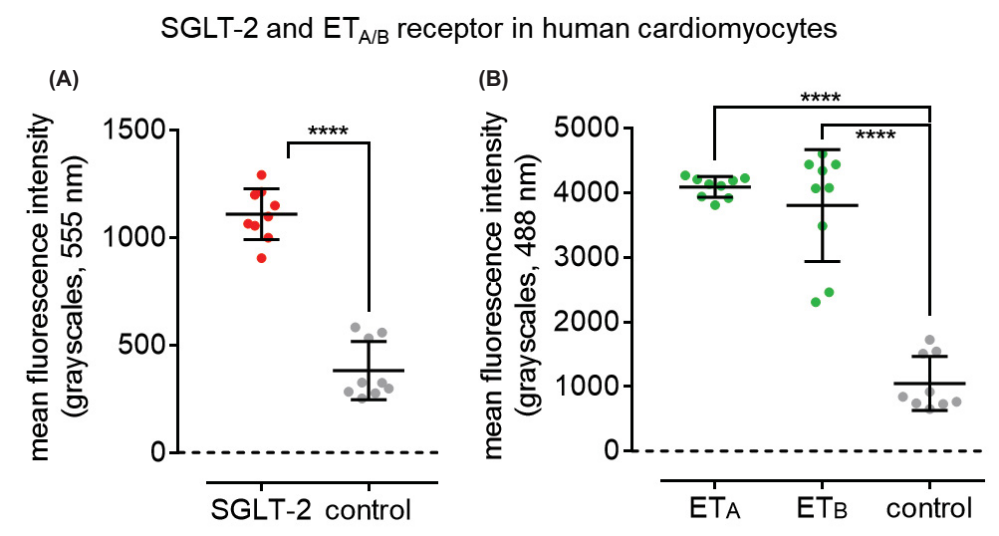
Quantification of SGLT-2, ET_A_ and ET_B_ immunofluorescence in human cardiomyocytes Bar chart shows quantitative 555 nm fluorescence (grayscales) for cardiac structures stained with SGLT-2 antisera (**A**) and quantitative 488 nm fluorescence (grayscales) for cardiac structures stained with ET_A_ or ET_B_ antisera (**B**) compared with control sections. Data are expressed as mean ± s.e.m.,* n* = 3 individuals, in triplicate. Statistical significance was determined using a one-way ANOVA, with Tukey’s correction for multiple comparisons looking for differences between treatment groups; ****P<0.0001.

Co-localization of ET_A_ receptor immunoreactivity with the specific smooth muscle cell marker, α-actin and ET_B_ with vWF, a specific marker of endothelial cells was confirmed in adjacent sections of left ventricle (Supplementary Figure S1) Quantification of SGLT-2, ET_A_ and ET_B_ immunofluorescence in human cardiomyocytes is shown in (Figure 4).

### Human heart control

**Figure 5:**
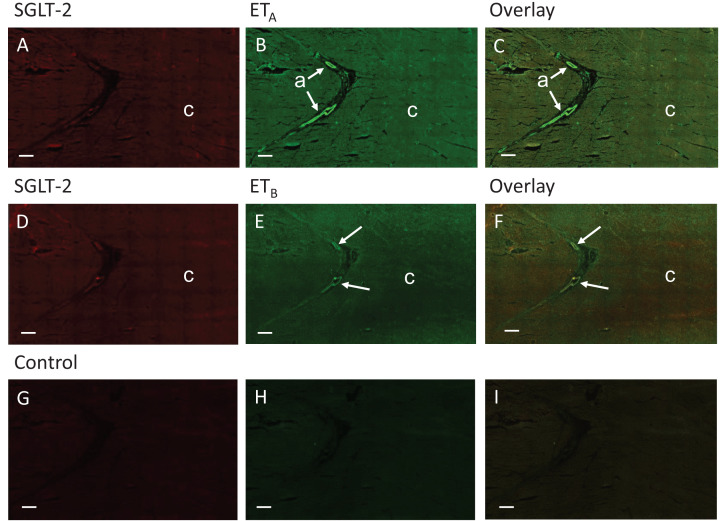
SGLT-2, ET_A_ and ET_B_ distribution in control human heart - left ventricle tissue Representative fluorescent images of sections of left ventricle from control following dual labelling antisera to SGLT-2 (**A,D**, red) and ET_A_ (**B**, green) or ET_B_ (**E**, green) with the overlay (**C** and **F**). The pattern was similar to DCM left ventricle. ET_A_ receptors on smooth muscle are shown in (B and C), arrows point to arteries (indicated with lower case a), c indicates examples of cardiomyocytes within the ventricular free wall. ET_B_ staining was detected in endothelial cells in E and F, indicated by arrows. (**G–I)** Adjacent sections where the primary antisera have been omitted to show levels of background fluorescence; scale bar = 50 µm.

Expression of SGLT-2, ET_A_ and ET_B_ receptors in the control heart was very similar to that described above in DCM heart (Figure 5).

### Human embryonic stem cell-derived cardiomyocytes

**Figure 6:**
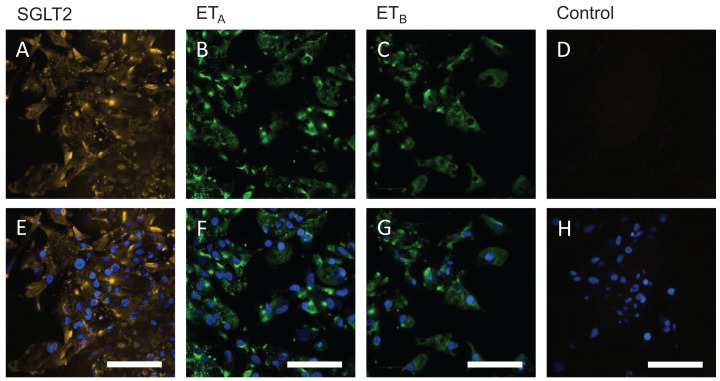
SGLT-2, ET_A_ and ET_B_ immunofluorescence staining in human embryonic stem cell-derived cardiomyocytes Representative fluorescent images of SGLT-2 immunoreactivity (gold) in beating hESC-CMs following dual labelling antisera to SGLT-2 (**A,E**, gold) and ET_A_ (**B,F**, green) or ET_B_ (**C,G**, green). For controls, primary antisera have been omitted (**D**). Lower panels (**E–H**) show the overlay with Hoechst 33342 nuclear stain; scale bar = 50 µm.

In hESC-CMs, punctate SGLT-2 immunoreactivity localised to the cytoplasm (Figure 6A) but not to the nucleus, visualised in the lower panel (Figure 6E) with nuclear staining (blue). This pattern of staining was consistent with primary cultures of renal proximal epithelial cells (Baer et al., 2020). ET_A_ (Figure 6B) and ET_B_ (Figure 6C) immunoreactivity was also localised to the cytoplasm as expected but not to the nuclei (Figure 6F,G).

## Discussion

In the kidney, co-localization of both ET receptor sub-types with SGLT-2 was restricted to epithelial cells of the proximal convoluted tubules [16]. mRNA encoding ET-1 together with both sub-types has previously been localised to these cells and expression of both sub-types can be increased in hypoxia [17]. The effect of SGLT-2i on the ET pathway has not been extensively studied. However, SGLT-2 inhibition *in vitro* has been shown to reduce both basal and stimulated ET-1 production in human proximal tubular cells [18]. Intriguingly, data from patients with heart failure demonstrates the beneficial effect of dapagliflozin is preserved amongst patients with high baseline plasma ET-1 levels and treatment resulted in a modest reduction in plasma ET-1 after 12 months. This interaction has not yet been investigated prospectively [19]. Our co-localization results support a potential new mechanism of action on the proximal tubules in reducing ET-1 and contributing to the renal benefit of SGLT-2 inhibition.

Punctate SGLT-2 protein localised to the cytoplasm but not within the nuclei in beating hESC-CMs, recapitulating the results in tissue sections of ventricle. This finding is consistent with the detection of mRNA encoding the transporter in cardiomyocytes derived from human induced pluripotent stem cells [10]. hESC-CMs are widely used as clinically relevant models to explore the physiology and pharmacology of therapeutic targets such as transporters and ion channels (see for example [10,20]) as well as phenotypic drug screens in genetically modified cells.

Multiple mechanisms of action for SGLT-2i remain under investigation to explain the efficacy demonstrated in heart failure. SGLT2i’s result in glycosuria and as a consequence osmotic diuresis. Diuresis is used in chronic heart failure to reduce the consequences of elevated venous pressure, such as congestive tissue oedema and excessive cardiac dilatation [7]. Also, relative hypoinsulinemia induces a favourable shift towards increased lipolysis and therefore increased circulating ketone bodies [21], a key source of energy for cardiomyocytes in heart failure [22]. Elevated circulating ketone bodies during SGLT-2 therapy in diabetic patients has been correlated with reduced activity of the pattern receptor recognition protein: the NLRP3 inflammasome complex in macrophages, which is a pathway of innate immunity important in the pathogenesis of atherosclerosis and coronary heart disease [23].

**Figure 7:**
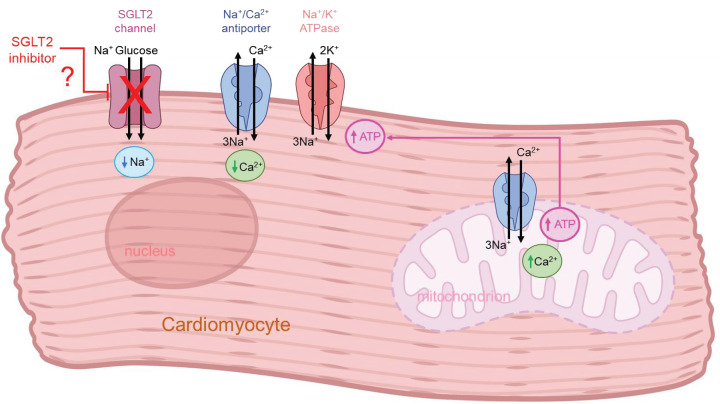
Schematic illustrating SGLT-2 interactions in cardiomyocytes Heart failure and type 2 diabetes result in detrimentally increased *cytoplasmic* Na^+^ and Ca^2+^ concentrations but reduced *mitochondrial* Ca^2+^ and ATP generation. The *cytoplasmic* Na^+^/Ca^2+^ antiporter 1 transports Na^+^ across the plasma membrane in exchange for a single Ca^2+^ moving in the opposite direction, the principal Ca^2+^ efflux mechanism, that is impaired by rising cytoplasmic Na^+^. It is speculated SGLT-2i could act directly on SGLT-2, to beneficially lower *cytoplasmic* Na+ and Ca^2+^. This reduced *cytoplasmic* Na+ results in reduced loss of Ca^2+^ from *mitochondrial* Na^+^/Ca^2+^ antiporter 1 to increase *mitochondrial* Ca^2+^, beneficially increasing ATP production, improving respiration. The *cytoplasmic* Na^+^ K^+^ ATPase pumps 3 Na^+^ out of the cell and 2K^+^ into the cell, for a single molecule of ATP and helps to maintain osmotic equilibrium and membrane potential in cells. Created with BioRender.com.

Additionally, direct effects of SGLT-2i on cardiomyocytes have been proposed [24] (Figure 7). SGLTi have been associated with positive effects on sodium and calcium homeostasis, in particular a reduction in cytoplasmic Na^+^ concentration, late I_Na_ current. Concomitant effects of reduced cytosolic Ca^2+^ concentration and increased mitochondrial Ca^2+^ have also been reported with empagliflozin [25]. Amongst other cellular benefits relevant for the progression of heart failure, these changes favour improved energy metabolism and anti-oxidant functions in mitochondria; however, detailed characterisation of the specific pathways remains lacking.

Expression of SGLT-2 receptors on cardiomyocytes has generated controversy. Dynamic changes of expression of SGLT-2 have been measured in a mouse model of myocardial infarction specifically in the infarcted zone [26]. Human studies have previously reported no expression of SGLT-2 on myocardial tissue [27,28]; however, this has been contradicted by a recent study in heart transplant patients, which also reported elevated cardiomyocyte SGLT-2 expression within specimens obtained from diabetic patients [8]. Our results support the presence of SGLT-2 immunoreactivity cardiomyocytes in left ventricle in patients with CDM as well as controls. Whether these low levels of SGLT-2 have a (patho)physiological role in cardiomyocytes is not yet known Our results suggest the effect of direct blockade of sodium (and glucose) influx via SGLT-2 inhibition in cardiomyocytes should be explored, with potential for additive actions with ET_A_ antagonists.

## Clinical perspectives

- ET receptor antagonists are being investigated in clinical trials in combination with sodium-glucose co-transporter-2 inhibitors (SGLT-2i). to primarily inhibit the SGLT-2 transporter that, in humans, is thought to be mainly restricted to the renal proximal convoluted tubule, resulting in increased glucose excretion favouring improved glycaemic control and diuresis. This action reduces fluid retention with ET receptor antagonists.
- SGLT-2 immunoreactivity localised as expected to epithelial cells of the proximal convoluted tubules in the kidney, and co-localised with both ET receptor sub-types. ET_A_ receptors predominated as expected from previous studies in cardiomyocytes and co-localised with lower (compared with epithelial cells) but positive staining for SGLT-2.
- The results suggest SGLT-2 may also have a physiological or pathophysiological role in cardiomyocytes. The effect of direct blockade of sodium (and glucose) influx via SGLT-2 inhibition into cardiomyocytes requires further investigation to established whether there is also a beneficial direct action on the heart and the potential for additive actions with ET_A_ antagonists.

## Supplementary Material

Supplementary Figure S1

## Data Availability

The data that support the findings of this study are available from the corresponding author upon reasonable request.

## Open Access

Open access for this article was enabled by the participation of University of Cambridge in an all-inclusive *Read & Publish* agreement with Portland Press and the Biochemical Society under a transformative agreement with JISC.

## Abbreviations

DCM: dilated cardiomyopathy
dct: distal convoluted tubules
ET: endothelin
ET_A_: endothelin ET_A_ receptor
ET_B_: endothelin ET_A_ receptor
g: glomeruli
H&L: heavy and light chain
hESC-CM: human embryonic stem-cell derived cardiomyocyte
LED: light-emitting diode
PBS: phosphate buffered saine
pct: proximal convoluted tubules
RNA-Seq: RNA sequencing
SGLT-2: sodium-glucose co-transporter-2 channel
SGLT-2i: sodium-glucose co-transporter-2 channel inhibitor
T2DM: Type 2 Diabetes
vWF: von Willebrand factor
