# Supplementary material for: Co-localization of the sodium-glucose co-transporter-2 channel (SGLT-2) with endothelin ETA and ETB receptors in human cardiorenal tissue
Source: Biosci Rep. 2024 May 31;44(6):BSR20240604. doi: 10.1042/BSR20240604 (PMC11147812; doi:10.1042/BSR20240604)
Supplement: Supplementary Figure S1 [file BSR-2024-0604_supp.pdf]

Supplementary Figure 1

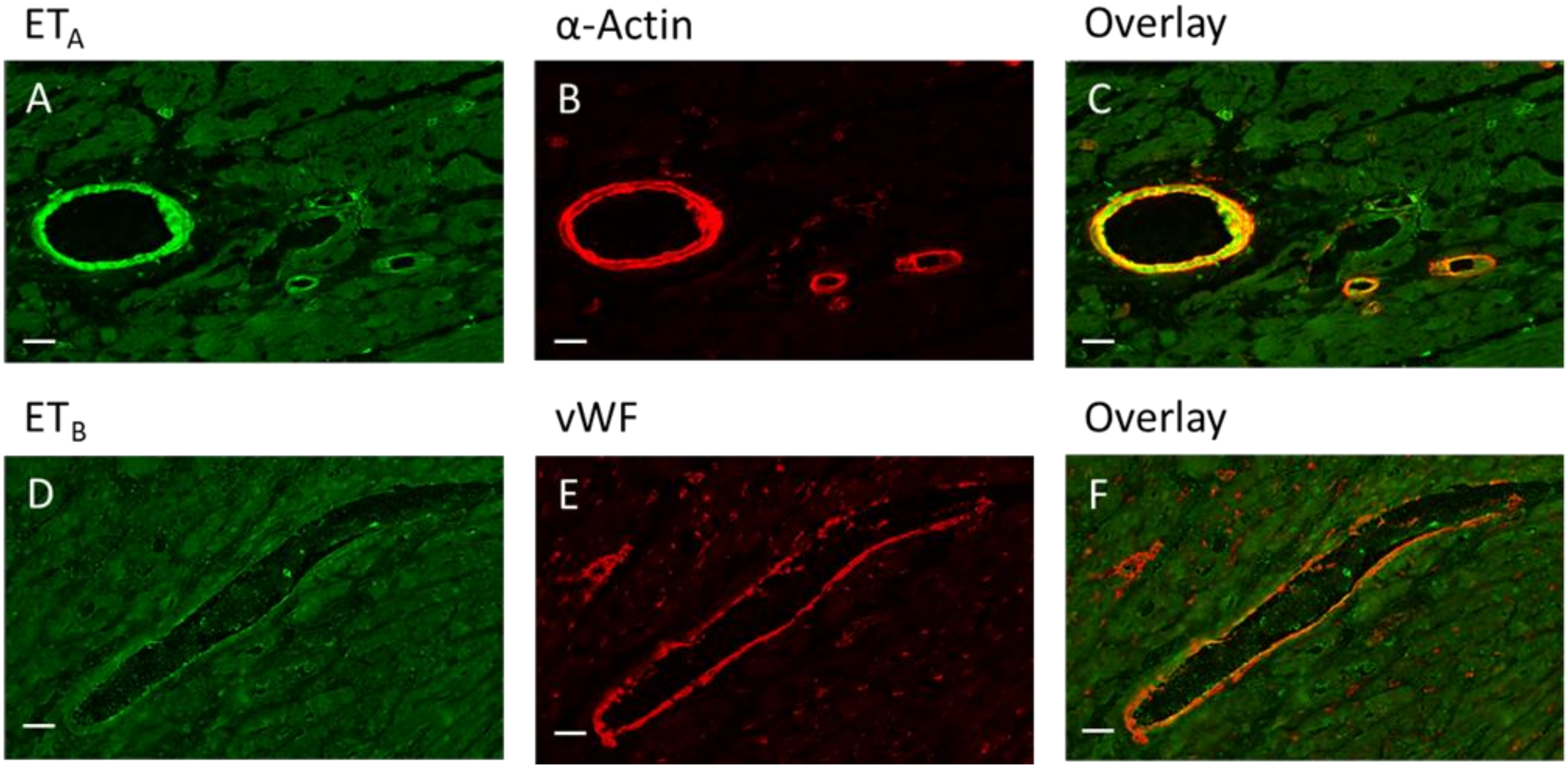

Supplementary Figure 1. ET_A and ET_B immunofluorescence staining in human heart left ventricle and blood vessels.

Representative fluorescent image of a section of left ventricle from DCM heart following dual labelling with antisera to ET_A receptor (A, green) showing colocalization with smooth muscle cell marker, α-actin (B, red) in a transverse section of a vessel. Scale bar = 50 µm.

A representative image following dual labelling of ET_B receptors (D, green), showing colocalization with to the endothelium, identified by the cell specific marker vWF (E, red) in a longitudinal section a vessel. Scale bar = 45 µm.
